# Surface modification and characterization of carbon spheres by grafting polyelectrolyte brushes

**DOI:** 10.1186/1556-276X-9-283

**Published:** 2014-06-04

**Authors:** Qi Zhang, Houbin Li, Pan Zhang, Liangliang Liu, Yuhang He, Yali Wang

**Affiliations:** 1 School of Printing and Packaging, Wuhan University, Wuhan 430079, People's Republic of China; 2 State Key Laboratory of Pulp and Paper Engineering, South China University of Technology, Guangzhou 510641, People's Republic of China

**Keywords:** Carbon spheres, Cation spherical polyelectrolyte brushes, Surface modification, Azo initiator, DMDAAC

## Abstract

Modified carbon spheres (CSPBs) were obtained by grafting poly(diallyl dimethyl ammonium chloride) (p-DMDAAC) on the surface of carbon spheres (CSs). It can be viewed as a kind of cation spherical polyelectrolyte brushes (CSPBs), which consist of carbon spheres as core and polyelectrolytes as shell. The method of synthesizing carbon spheres was hydrothermal reaction. Before the polyelectrolyte brushes were grafted, azo initiator [4,4′-Azobis(4-cyanovaleric acyl chloride)] was attached to the carbon spheres' surface through hydroxyl groups. CSPBs were characterized by scanning electron microscope (SEM), Fourier transform infrared spectroscopy (FTIR), gel permeation chromatography (GPC), conductivity meter, and system zeta potential. The results showed that compared with carbon spheres, the conductivity and zeta potential on CSPBs increased from 9.98 to 49.24 μS/cm and 11.6 to 42.5 mV, respectively, after the polyelectrolyte brushes were grafted. The colloidal stability in water was enhanced, and at the same time, the average diameter of the CSPBs was found to be 173 nm, and the average molecular weight and grafted density of the grafted polyelectrolyte brushes were 780,138 g/mol and 4.026 × 10^9^/nm^2,^ respectively.

## Background

Owing to their particular physical and chemical properties, carbon spheres (CSs) have attracted much attention of many researchers in different areas [1]. Because of their porous structure [2], high surface area, high electrical conductivity, thermal stability [3], and excellent chemical stability, CSs have been widely used as anode material for lithium-ion battery [4], cathode materials for field emission [5], catalyst support materials [6], and adsorbents [7]. But the poor dispersibility of CSs in water has greatly affected their further application in aqueous phase field, such as biological medicine, wastewater treatment, and catalysis. In order to improve the dispersibility in water, many researchers have changed the surface modification of carbon spheres by using air oxidation and mixed acid oxidation. Zhang and colleagues [8] used phosphate group to increase the content of oxygen-containing functional groups on the surface of phosphorus-rich hydrothermal carbon spheres. Researchers [9] in Anhui Key Laboratory of Advanced Building Materials added ammonia to hydrothermal reaction solution to get carbon spheres with amino groups, which showed an excellent enhanced adsorption performance for the removal of heavy metal anions. Liu et al. [10] introduced functional double bonds onto the surface of CSs by covalent and non-covalent method to improve CSs' dispersibility and compatibility in polymer matrix, in which covalent functionalization was accomplished through mixed acid oxidation and subsequent reaction with acryloyl chloride. Lian et al. [11] modified polystyrene-based activated carbon spheres with either air, HNO_3_, (NH_4_)_2_S_2_O_8_, H_2_O_2,_ or H_2_ to improve their adsorption properties of dibenzothiophene. Although many researches have been done to modify the surface of CSs, there was still potential damage to the structure of carbon materials [12].

In this paper, the method of grafting polyelectrolyte brushes on the surface of CSs was used to enhance the dispersibility of CSs in water. First, the CSs were prepared by hydrothermal reaction solution. Then, the process of grafting polyelectrolyte brushes was conducted on the surface of the CSs. The method of preparing CSs with hydrothermal reaction solution was environmental, simple, and can be easily controlled, and there were much more hydroxyl groups that could be obtained on the surface of CSs than any other methods. Compared with air oxidation and mixed acid oxidation, the modification by grafting polyelectrolyte brushes on the surface of CSs would not influence the inner structure of CSs at all, and it could not only protect the original properties of CSs but also enable CSs to have some new and different properties because of the variability of kinds of polyelectrolyte brushes. In this paper, poly(diallyl dimethyl ammonium chloride) (p-DMDAAC) has been chosen as the polyelectrolyte brush. After being grafted, CSs became more stable in water than before.

## Methods

### Raw materials and reagents

The chemicals used in this study are the following: glucose (Guoyao Group of Chemical Reagents Ltd., Shanghai, China), 4,4′-Azobis (4-cyanovaleric acid) (ACVA; Aladdin Company, Shanghai, China), diallyl dimethyl ammonium chloride (DMDAAC; Aladdin Company, Shanghai, China), dichloromethane (Guoyao Group of Chemical Reagents Ltd.), hexane (Guoyao Group of Chemical Reagents Ltd.), ethanol, toluene, triethylamine, distilled water, and phosphorus pentachloride. All the chemicals and solvents used in this study were of analytical grade.

### Synthesis of carbon spheres

Glucose solution was used as a carbon source to make CSs in an autoclave. The process required 6 h at 180°C [13].

### Synthesis of azo initiator (4,4′-Azobis (4-cyanovaleric acyl chloride))

ACVA (1.4 g) was dissolved in 40 ml dichloromethane. About 9 g of PCl_5_ was taken in 50 ml dichloromethane. Then, the ACVA solution was added to the reaction mixture. Throughout the reaction, the temperature was maintained below 10°C [14]. The reaction mixture was kept for 48 h under nitrogen atmosphere. The purified product was obtained by rotary evaporation and extraction with hexane.

### Immobilized ACVC on CSs

**Figure 1:**
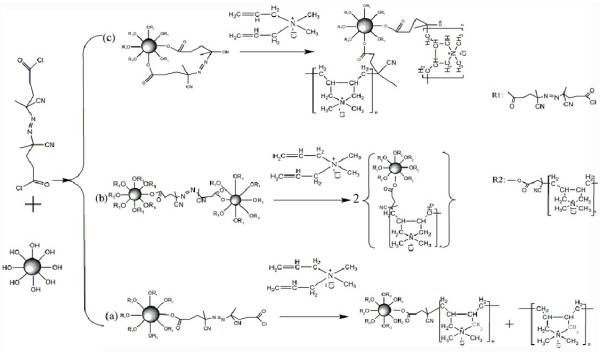
**Modification process of carbon spheres. (a)** Single-ended form grafted on CSs, **(b)** double-ended form grafted on hetero-CSs, and **(c)**  double-ended form grafted on homo-CSs.

The schematic diagram of the synthesis process of CSs immobilized with ACVC is shown in Figure 1. About 0.4 g CSs was put in 10 ml anhydrous toluene; 3 ml triethylamine was added as catalyst. About 3.17 g ACVC was dissolved in 30 ml anhydrous toluene. Then, the ACVC solution was added drop by drop to the reaction mixture and kept for 24 h with stirring at room temperature under nitrogen atmosphere. After the reaction, the crude product was washed by toluene and dried under vacuum for 24 h at 25°C to obtain the purified product (CSs-ACVC).

### Surface modification of CSs by grafting polyelectrolyte brushes

A certain amount of CSs-ACVC, a solution of diallyl dimethyl ammonium chloride, and distilled water (1/1 *v*/*v*) were put in a flask. Ultrasonic treatment was used to ensure that the mixture solution is dispersing uniformly. Then, the system was carefully degassed to remove the oxygen in 30 m and then the polymerization from the surface of CSs-ACVC was carried out at 60°C. Within 9 h, cation spherical polyelectrolyte brushes (CSPBs) were obtained. To gain pure CSPBs, the product was purified with distilled water by Soxhlet extraction. The substance existing in the washing liquor of CSPBs was testified to be p-DMDAAC. Because the weight-average molecular weight of the washing liquor of CSPBs was equal to that of p-DMDAAC grafted on the surface of CSs (p-DMDAAC-CSs), p-DMDAAC in washing liquor of CSPBs (p-DMDAAC-WL) can be collected to characterize the weight-average molecular weight of p-DMDAAC-CSs.

### Characterization

When Fourier transform infrared spectroscopy (FTIR) (Nicolet AVATAR 360FT, Tokyo, Japan) was used to analyze the structure of the obtained products, the morphology of the CSPBs was characterized by scanning electron microscope (SEM) (Quanta 200, Holland, Netherlands). The weight of p-DMDAAC-CSs was calculated by thermogravimetric analysis (TGA) (SETSYS-1750, AETARAM Instrumentation, Caluire, France). The weight-average molecular weight of p-DMDAAC-CSs was determined by gel permeation chromatography (GPC) (Waters 2410 Refractive Index Detector, Waters Corp., Milford, MA, USA). The conductive performance of CSs and CSPBs was tested by a conductivity meter (DDS-12DW Microprocessor Conductivity Meter, A&E Laboratory Co. Ltd., Guangdong, China). Zeta potential on CSs and CSPBs was tested by system zeta potential (Zetasizer Nano-ZS, Malvern Instruments Ltd., Malvern, UK).

## Results and discussion

### Morphology analysis

**Figure 2:**
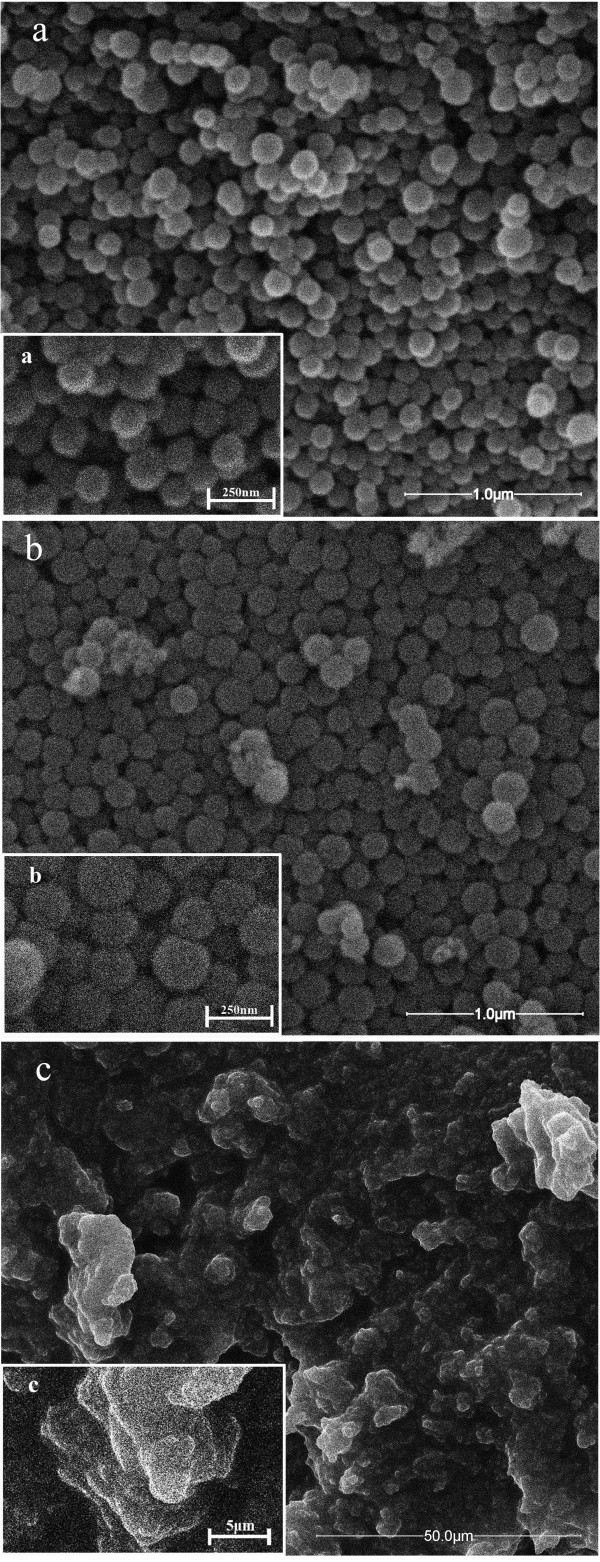
**SEM photographs. (a)** CSs, **(b)** CSPBs, and **(c)**  p-DMDAAC-WL.

The morphologies of CSs, CSPBs, and p-DMDAAC-WL are displayed in Figure 2a,b,c, respectively. The average diameter of CSPBs was 173 nm, larger than that of CSs (153 nm). It indicated that there were indeed some polymer brushes on the CSs' surface. As shown in Figure 1, there existed three kinds of patterns for this polymerization. If the reaction occurred as route b or c, there would be no polymer appearing in the washing liquor of the CSPBs. However, from Figure 2c, bulk polymer (p-DMDAAC-WL) has been seen obviously. Thus, it can be confirmed that in the synthesis of immobilizing ACVC on CSs, the main products obtained were in the single-ended form grafted on CSs (see Figure 1a). Owing to the breaking of the azo linkage, half of the initiator was detached from the surface of the CSs, which induced homopolymerization of DMDAAC.

### FTIR analysis

**Figure 3:**
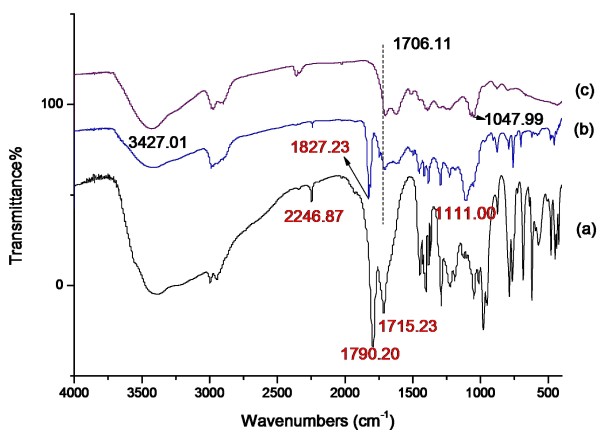
**FTIR spectra. (a)** ACVC, **(b)** ACVC immobilized on CSs, and **(c)** CSs.

The successful synthesis of 4,4′-Azobis (4-cyanovaleric acyl chloride) was testified by FTIR (see Figure 3 spectrum a). The vibration absorption peaks of -COCl (at 1,790 cm^-1^) and -C ≡ N (at 2,246 cm^-1^) were observed obviously. The FTIR spectrum of CSs (see Figure 3 spectrum c) showed strong vibration absorption peaks of -OH (at 3,427 cm^-1^). A new peak in the FTIR spectrum of CSs immobilizing with ACVC (see Figure 3 spectrum b) indicated that CSs induced redshift of the vibration absorption of -COCl, jumping from 1,790 to 1,827 cm^-1^. The peak at 1,111 cm^-1^ represented -C-O-C- for CSs immobilizing with ACVC.

### Thermal stability

**Figure 4:**
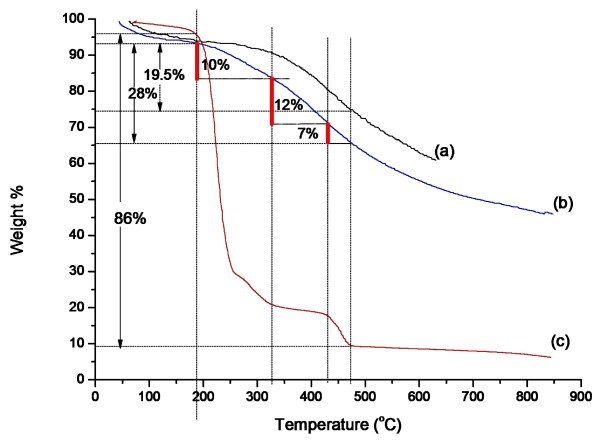
**Thermography curves. (a)** Pure CSs, **(b)** CSPBs, and **(c)** p-DMDAAC-WL.

Because it is difficult to calculate the weight of p-DMDAAC-CSs, thermogravimetry analysis of CSs, CSPBs, and p-DMDAAC-WL has been done, respectively, to distinguish the proportion of CSs and p-DMDAAC in CSPBs. As shown in Figure 4, the mass loss below 190°C shown in all these three curves implied a loss of moisture. From the curve of p-DMDAAC-CSs (see Figure 4 curve c), it could be ensured that the washing liquor of CSPBs was p-DMDAAC [15]. As shown in Figure 4 curve b, the mass loss (10%) from 190°C to 330°C was mainly the decomposition of p-DMDAAC-CSs. And the stage from 330°C to 430°C mainly implied the loss of CSs (12%). During the period from 430°C to 475°C, mass loss contains both CSs and p-DMDAAC-CSs (7%).

### Calculation of surface grafting density

As shown in Figure 4 curve b, the weight loss (28%) from 190°C to 475°C contained the decomposition of both CSs and p-DMDAAC-CSs. The weight loss of CSs and p-DMDAAC-CSs during the same period was 19.5% and 86%, respectively (as shown in Figure 4 curves a and c). The weight-average molecular weight of p-DMDAAC-WL was 780,138 g/mol tested by GPC. The weight of p-DMDAAC-CSs (*m*) could be calculated according to formula (1). The percentage of the grafted p-DMDAAC-CSs and surface grafting density (*σ*) were calculated according to formula (2).

(1) $$m=m_{0}\left(w_{0}\%-w_{1}\%\right)/\left(w\%-w_{1}\%\right),$$

where *m*_0_ is the weight of the CSPBs used for TGA, *w*_0_% is the weight loss of the CSPBs during the temperature rise from 190°C to 475°C, *w*_1_% is the mass loss of the pure CSs in the same temperature, and *w*% stood for the mass loss of p-DMDAAC-WL.

(2) $$\sigma =m\times \mathrm{NA}/\mathrm{Mw}\times 4\pi r^{2},$$

where *Mw* is the weight-average molecular weight of p-DMDAAC-CSs, and *r* is the average size of the CSs.

### Conductivity tests

**Figure 5:**
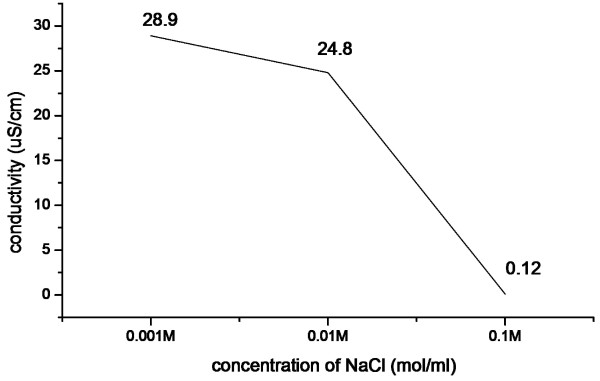
Conductivity of CSPBs in different concentrations of NaCl.

Conductivity has been tested to compare the promotion of conductive performance of CSs and CSPBs. A 1.5-mg/ml solution of CSs and CSPBs was prepared with water as solvent. The conductivity of CSs and CSPBs water solution was 9.98 and 49.24 μS/cm, respectively. It can be turned out that the conductivity of CSs increased with the grafting of p-DMDAAC on the surface of the CSs. As shown in Figure 5, the conductive performance of CSPBs decreased with the increase of ionic strength by adding the amount of salt. The reason for this phenomenon was that with the increasing ionic strength, the Debye length diminished [16], inducing the decreasing of the points on the polyelectrolyte brushes.

### Zeta potential and colloidal stability analysis

**Figure 6:**
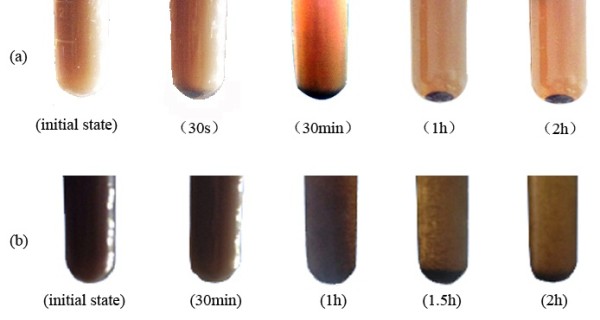
Dispersibility of (a) CSs and (b) CSPBs at different times in water.

The zeta potential on the CSs and CSPBs was 11.6 and 42.5 mV, respectively. It showed that polyelectrolyte was successfully grafted on the CSs. And the increase gained in the aspect of zeta potential enabled CSPBs to have better stability in water. As shown in Figure 6, the stratification of CSs appeared 30 s after ultrasonic dispersion, while the CSPBs appeared 1 h later.

## Conclusion

Surface modification of carbon spheres by grafting p-DMDAAC on their surfaces has been described, and a series of characterization was done. Using FTIR, SEM, conductivity meter, and zeta potential method, the chemical structure, morphology, conductivity, and water dispersibility of the modified CSs were represented. Owing to the p-DMDAAC-CSs, the dispersibility of CSPBs in water has been enhanced obviously, which will expand its application in liquor phase. Because the weight-average molecular weight and surface grafting density can be controlled by adjusting monomer concentration and reaction time, CSPBs with different performances will be obtained; thus, this will further expand its application field.

## Abbreviations

ACVA: 4,4′-azobis-(4-cyanovaleric acid); ACVC: azo initiator (4,4′-Azobis-(4-cyanovaleric acyl chloride)); CSPBs: modified carbon spheres; CSs: carbon nanospheres; CSs-ACVC: carbon spheres immobilized with 4,4-Azobis-(4-cyanovaleric acid); DMDAAC: diallyl dimethyl ammonium chloride; FTIR: Fourier transform infrared spectroscopy; GPC: gel permeation chromatography; p-DMDAAC: poly(diallyl dimethyl ammonium chloride); p-DMDAAC-CSs: p-DMDAAC grafted from the surface of CSs; p-DMDAAC-WL: p-DMDAAC in washing liquor of CSPBs; SEM: scanning electron microscope; TGA: thermogravimetric analysis.

## Competing interests

The authors declare that they have no competing interests.

## Authors' contributions

HL made substantial contributions to the conception, design, and supervision of the whole study. QZ carried out the whole modification of the CSs and drafted the manuscript. YW and PZ carried out the characterization measurements. LL and YH contributed to the analysis and interpretation of the data. All authors read and approved the final manuscript.

## Authors' information

HL is a professor in the School of Printing and Packing at Wuhan University, China. He is a Ph.D. supervisor. His main research interests include packing materials, packing auxiliary materials, and printing materials. QZ, PZ, and YW are studying for a masters degree at Wuhan University. QZ's research subject is related to the use of polyelectrolyte brushes to achieve surface modification of carbon nanospheres. Selecting modified carbon nanospheres as retention and drainage agents and applying them to the papermaking industry is the next research work of QZ. LL has graduated from Wuhan University. Currently, he works in Haosen Packaging Company, China. YH is currently doing his Ph.D. in the School of Printing and Packing at Wuhan University. He did his M.Sc. in the College of Chemistry Molecular Science at Wuhan University. His research focus is on polyelectrolyte brushes.
